# Band-structure-engineered high-gain LWIR photodetector based on a type-II superlattice

**DOI:** 10.1038/s41377-020-00453-x

**Published:** 2021-01-14

**Authors:** Arash Dehzangi, Jiakai Li, Manijeh Razeghi

**Affiliations:** Center for Quantum Devices, Department of Electrical and Computer Engineering, Northwestern University, Evanston, IL 60208 USA

**Keywords:** Photonic devices, Optics and photonics

## Abstract

The LWIR and longer wavelength regions are of particular interest for new developments and new approaches to realizing long-wavelength infrared (LWIR) photodetectors with high detectivity and high responsivity. These photodetectors are highly desirable for applications such as infrared earth science and astronomy, remote sensing, optical communication, and thermal and medical imaging. Here, we report the design, growth, and characterization of a high-gain band-structure-engineered LWIR heterojunction phototransistor based on type-II superlattices. The 1/*e* cut-off wavelength of the device is 8.0 µm. At 77 K, unity optical gain occurs at a 90 mV applied bias with a dark current density of 3.2 × 10^−7^ A/cm^2^. The optical gain of the device at 77 K saturates at a value of 276 at an applied bias of 220 mV. This saturation corresponds to a responsivity of 1284 A/W and a specific detectivity of 2.34 × 10^13^ cm Hz^1/2^/W at a peak detection wavelength of ~6.8 µm. The type-II superlattice-based high-gain LWIR device shows the possibility of designing the high-performance gain-based LWIR photodetectors by implementing the band structure engineering approach.

Recent advances in semiconductor materials and devices have led to much progress in the development of photodetectors for numerous applications across a variety of fields. Photodetectors are now able to broadly cover wavelengths from deep UV, visible, and near-infrared spectra all the way up to long-wavelength infrared (LWIR) and even terahertz spectral bands^1–8^. As the prospects for conventional *pin* detectors begin to saturate, there is a need to develop new designs, such as barrier photodetectors and ultra-sensitive devices with internal/intrinsic gain, that can yield better detectivity.

The current state-of-the-art LWIR detection technology is based on mercury cadmium telluride (HgCdTe) materials, which can achieve excellent sensitivity and speed. This material has been used to realize LWIR avalanche photodiodes (APDs) based on the gain from impact ionization mechanisms^9,10^; however, in general, APD structures suffer from low photocurrent gain, which requires high bias voltages and suffer from excess noise associated with the avalanche multiplication process. Additionally, the Hg_*x*_Cd_1−*x*_Te compositions needed for LWIR detectors are difficult to grow consistently, which leads to poor spatial uniformity. This complex material growth, as well as the additional challenges associated with processing II–VI materials, reduces device fabrication yield and significantly increases production costs^11^.

Alternate commercial technologies in the LWIR band include photodetectors based on vanadium oxide or amorphous silicon (α-Si), which offer several benefits such as high-temperature operation, compatibility with complementary metal–oxide–semiconductor technology, and low fabrication costs. However, applications for these photodetectors are limited by shortcomings such as limited tunability of the detection wavelength, poor sensitivity, and slow response speed^12^.

LWIR photodetectors based on graphene, or another material in combination with graphene, are being developed as a potential solution for future high-performance photodetectors^13–15^. However, the application of these graphene-based devices is limited by the vanishing bandgap and poor light absorption of thin graphene layers, which in turn increases the dark current and overall noise level. Although approaches are being developed to improve the performance of graphene-based photodetectors, such as surface plasma-enhanced light absorption and carrier multiplication^16,17^, these graphene-based LWIR photodetectors still require additional development before they can become competitive.

Beyond graphene, recent progress in other two-dimensional materials (2DMs) has revealed interesting results for gain-based mid-wavelength infrared (MWIR) and LWIR photodetectors. High-gain MWIR black phosphorus (b-P) has demonstrated high responsivity, but this was at the expense of a much slower speed^18,19^. In addition, there has been difficulty achieving detection at wavelengths longer than 7 μm^20^. Newly developed black arsenic phosphorus (b-AsP) has a bandgap that can be adjusted for detection near the LWIR atmospheric transmission window, and by increasing the arsenic mole fraction, this technology can demonstrate LWIR detection at room temperature^21,22^. Unfortunately, b-P and b-AsP are air-sensitive, which greatly complicates device fabrication and testing^23^. The responsivity of b-P and b-AsP devices is also lower than that expected for gain-based LWIR photodetectors. The highest responsivity of LWIR photodetection has been demonstrated using group X transition-metal dichalcogenides known as 2DMs, such as platinum diselenide and palladium diselenide^24,25^. Despite recent progress, several challenges must be overcome before 2DM can achieve commercial success as a viable alternative in the LWIR region. One major challenge for all 2DMs is the low absorption cross-section, which significantly limits the quantum efficiency (QE) and ultimately the responsivity. While 2DM photodetectors can partially compensate for this low QE with photoconductive gain, this tends to result in detectors with high dark current, high noise, and low detectivity^26^. Furthermore, the optical/electrical properties of these thin materials are strongly influenced and degraded by environmental circumstances^26,27^, which makes large-scale fabrication difficult, putting them far away from any level of manufacturing readiness at this time^28^.

One promising alternative to APDs for achieving high optical conversion gain is to integrate existing detectors with heterojunction bipolar transistors to create a heterojunction phototransistor (HPT)^29,30^. Several HPT devices have been reported based on compound semiconductor material systems (such as InGaAs, InGaP, InP, and SiGe)^31–35^; these HPT devices exhibit large internal gain with high stability, and unlike APDs, they have the ability to operate at low bias voltages^29,36,37^. However, these materials are limited to shortwave and near-infrared detection. One promising material that has shown the potential to push HPTs into the LWIR spectrum is type-II superlattices (T2SLs); HPT devices have already been reported that cover the extended short-wavelength and MWIR bands^38,39^.

T2SLs^40,41^ are a developing material system with outstanding growth uniformity^42^ and exceptional band structure engineering capabilities^43,44^ that have proven T2SLs to be a versatile candidate for enhancing infrared detection and imaging^41^ from the near-infrared to the LWIR and even towards the very-LWIR^45–48^.

In this letter, we use the HPT structure and band-structure engineering capabilities of InAs/GaSb and InAs/GaSb/AlSb/GaSb T2SLs to realize an LWIR photodetector. The exceptional band structure engineering capabilities of the T2SL material system allow each part of the device to be carefully tuned to use phototransistor action to achieve high optical gain, low noise, and high detectivity^49^.

The high-gain LWIR photodetector device structure (Fig. 1) is based on an *npn* structure with a narrow-bandgap InAs/GaSb *n-type* LWIR collector, a narrow-bandgap InAs/GaSb *p-type* base, and a wide-bandgap InAs/GaSb/AlSb/GaSb *n-type* hybrid emitter. The empirical tight-binding method (ETBM) was used to design the constituent layers and calculate the band offsets (Fig. 2b). A schematic diagram of the conduction (*E*_C_) and valence (*E*_V_) bands of the LWIR photodetector device structure is presented in Fig. 2a, where sectors 1–7 are the different segments of the structure. The bandgaps of the sectors are summarized in Table 1.

**Fig. 1:**
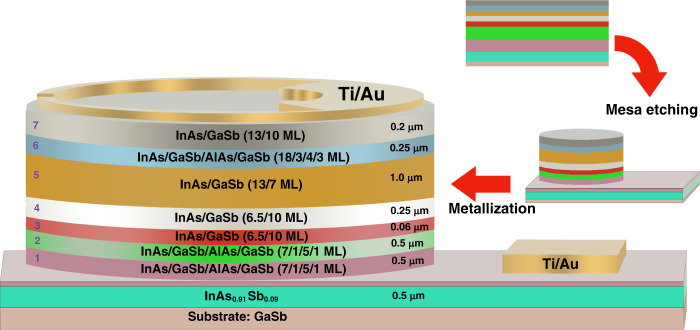
Schematic diagram of the LWIR T2SL phototransistor structure with a summary of the key fabrication steps at the right. Blue numbers indicate the sector designations used in Fig. 2, with 1 the bottom contact, 2 the emitter, 3 the base, and 4–5 the hybrid collector

**Fig. 2:**
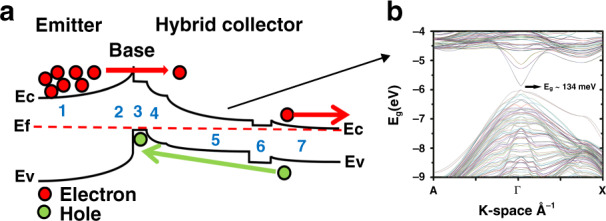
Energy band diagram and band structure of the HPT LWIR phototransistor device. **a** Schematic diagram of the conduction (*E*_C_) and valence (*E*_V_) bands of blue numbers 1–7 indicating the sectors of the device. **b** Band structure of the LWIR collector (sector 5) around the Γ-point calculated from the ETBM simulation with a bandgap of ~134meV

**Table 1:** The bandgap of each sector as calculated by the ETBM

Sector	1–2	3	4	5	6	7
Bandgap	635 meV	310 meV	310 meV	134 meV	166 meV	140 meV

The 60 nm thick p-type base region (sector 3) is placed between the bottom *n-contact*/emitter (sectors 1 and 2) as the electron injector and the hybrid collector (sectors 4 and 5). The n-type emitter consists of a T2SL with 7/1/5/1 monolayers (MLs) of InAs/GaSb/AlSb/GaSb, respectively. The strain is controlled by using binary InSb for one interface and InGaSb for the other. The narrow-bandgap p-type base consists of 6.5/10 MLs of InAs/GaSb with a bandgap of ~310 meV. The emitter has a much wider (~635 meV) bandgap with a precisely engineered band offset to the *p-type* base regardless of the doping concentration; using the ETBM, the emitter-base valence band offset was calculated to be ~303 meV. This heterojunction between the emitter and base (Fig. 2) permits sufficient injection efficiency (high optical gain) from the emitter to the base while blocking reverse carrier injection from the base into the emitter.

The abrupt junction between the narrow-band *p-doped* base and the narrow-band *n-doped* collector could trigger leakage current due to the reduction in the depletion width at this p-n junction, leading to enhancement of the electric field and causing band-to-band tunneling. To address this issue, an *n-type* hybrid T2SL-based collector structure was designed using the ETBM. Abutting the base is a lightly doped (*n-type*) 250 nm thick region (sector 4) consisting of 6.5/10 MLs of InAs/GaSb with the same 310 meV bandgap as in the base. There is then a 1.0 µm thick n-doped narrow-bandgap LWIR part (sector 5) with 13/6 MLs of InAs/GaSb T2SL and a bandgap of ~134 meV. The full band structure of this LWIR absorption region, as simulated by the ETBM, is shown in Fig. 2b.

The last part of the design is the electron extractor region (sectors 6 and 7). Using the band structure engineering capabilities of T2SL, this extractor creates a barrier in the valence band embedded between the collector and top n-contact. This hole barrier (sector 6) is 250 nm thick and consists of a T2SL with 18/3/4/3 MLs of InAs/GaSb/AlSb/GaSb; it has a bandgap of 166 meV with a valence band offset of ~100 meV with respect to the LWIR collector. Sector 7 is then a 200 nm thick top highly doped n-contact that will extract all the electrons and has a structure with 13/7 MLs of InAs/GaSb and a bandgap of 140 meV. The combination of sectors 6 and 7 allows efficient extraction of the electrons while blocking the transport of holes into the *n*-contact.

This device is designed to have a floating base where the photodetector absorption region is the collector, which is coupled directly to the base without the need for external contact to bias the base. This floating base design simplifies the fabrication of the device and, more importantly, permits direct illumination of the collector. Incident LWIR light is absorbed in the LWIR portion of the collector (sector 5), leading to the generation of electron/hole pairs. These carriers are then separated according to their minority carrier diffusion lengths in the collector and base and then swept away by the built-in electric field at the base–collector junction.

Because this design uses a much wider bandgap material for the emitter, there is a potential barrier in the valence band at the emitter-base junction (sectors 2 and 3) that serves to stop the generated holes from entering the emitter. Holes, therefore, accumulate in the base region, modulating the base potential and thereby forward biasing the emitter–base junction. This, in turn, modulates the electron barrier between the emitter and base, which allows electrons from the emitter to overpass the base region and enter the collector^29^. The use of a wide-bandgap emitter can provide an emitter–base injection efficiency close to unity, regardless of the relative base/emitter doping levels, since the valence band barrier effectively prevents hole injection from the base to the emitter^29^. The injected electrons coming from the emitter can preserve the charge neutrality in the base region and maintain the large electron current flow toward the collector, which is the source of the transistor action and high gain of this LWIR photodetector.

After fabrication, the devices were optically characterized via top illumination with either a Bruker IFS 66 v/S Fourier transform infrared spectrometer (FTIR) or a calibrated 1000 °C blackbody source and band-pass filter. In general, the responsivity of a detector is the ratio of the output electrical power over the input optical signal^50^. The responsivity can determine the optical efficiency on a macroscopic scale of power. The FTIR was used to measure the relative spectral response, and the absolute responsivity was calculated using a calibrated blackbody source and band-pass filter. No anti-reflection coatings were applied to the devices. All measurements were done at 77 K. Under 220 mV of applied bias, the responsivity reached a peak value of 1284 A/W at ~6.8 µm (Fig. 3a). The 1/*e* cut-off wavelength of the device was 8.0 µm at 77 K. At applied biases (*V*_b_) less than 220 mV, the responsivity at 6.8 µm decreased (Fig. 3b), and above 220 mV, the responsivity saturated. This strong dependence of the responsivity on the applied bias was related to the recombination rate in the base. At lower biases, electrons injected from the emitter spent more time in the base, which increased the probability that they would recombine with the holes that modulated the base transconductance—this amplified the effect of recombination in the base. Increasing the applied bias increased the electric field in the base region, leading to a higher drift velocity and a lower probability of recombination and thus a longer lifetime of the holes that modulate the transconductance of the base and an overall higher gain.

**Fig. 3:**
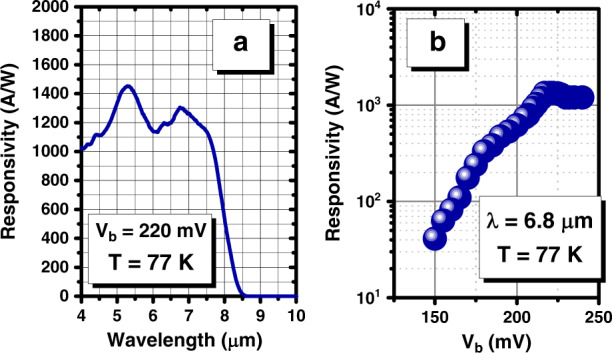
Optical performance of the LWIR T2SL phototransistor. **a** Saturated responsivity spectra of the device at 77K and *V*_b_=220mV. **b** The variation in the responsivity at 6.8µm versus the applied bias voltage (*V*_b_)

A 77 K cold shield was installed in a cryostat, and the dark current density and differential resistance-area product (R*A) versus *V*_b_ of a 200 µm diameter device were measured at 77 K (Fig. 4). The differential resistance-area product at zero bias was 1.2 × 10^7^ Ω cm^2^. Unity optical gain occurred at an applied bias of 129 mV, at which point the dark current density was 1.79 × 10^−6^ A/cm^2^. By 220 mV, when the responsivity saturated, the dark current density had increased to 3.2 × 10^−3^ A/cm^2^. The variation in the photocurrent generation at different temperatures is shown in Fig. 4c. The photocurrent follows an exponential trend and saturates in the range of ~10^−3^ A/cm^2^. Illumination was performed using a helium–neon (HeNe) laser with 5.0 mW of power.

**Fig. 4:**
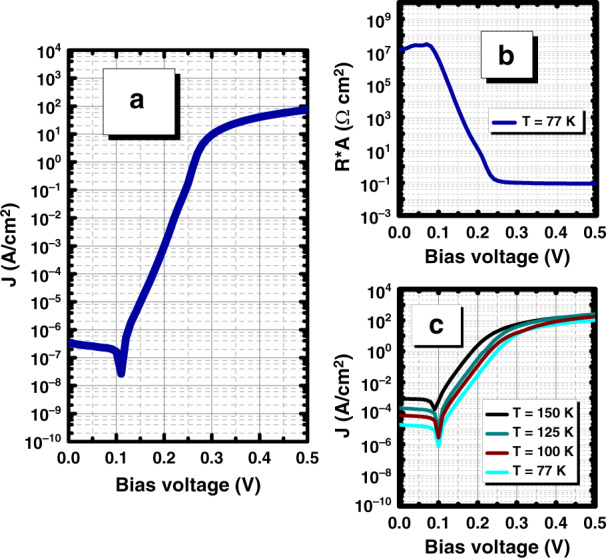
Electrical performance of the LWIR T2SL phototransistor. **a** Dark current density curves of the photodetector; **b** differential resistance-area product (R*A) at 77K vs. the applied bias voltage (*V*_b_); and **c** variation in the photocurrent generation at different temperatures (77–150 K)

The DC current gain (*β*) of the device was experimentally calculated via comparison to a reference sample. The reference LWIR sample was grown by molecular beam epitaxy (MBE) and processed in the same way as shown in Figs. 1 and 2, but the layer sequence omitted the emitter/injector part (sectors 1 and 2) to prevent the phototransistor gain. This reference device at 77 K exhibits a saturated responsivity of 1.54 A/W at 6.8 μm, which corresponds to an external QE of *η* = 0.35. Dividing the saturated responsivity of the phototransistors by the saturated responsivity of the reference sample yields an experimentally estimated DC current gain (*β*) of 833.

The optical gain (*O*_ptG_) of the device was also experimentally calculated directly from the saturated 6.8 µm responsivity measurements and from the calculation of the flux from the 1000 °C blackbody source. *O*_ptG_ is defined as the ratio of the number of carriers generated in the photocurrent to the number of coming incident photons. In practice, this approach means that we assume that the QE *η* = 1.0 (which is significantly higher than the *η* = 0.35 estimated from the reference device) and then simply normalize the responsivity to calculate the optical gain. The optical gain is directly proportional to the applied bias and increases with increasing *V*_b_. The *O*_ptG_ of the device saturates at a value of 276 at *V*_b_ greater than 220 mV and decreases to unity at *V*_b_ = 90 mV.

To better understand the potential for low-light operation, the optical gain at 6.8 µm was measured at various incoming optical powers spanning over 4 decades (Fig. 5). To achieve this measurement, the device bias was maintained at *V*_b_ = 220 mV (saturated responsivity), and the blackbody temperature was changed from 1000 °C down to 100 °C to vary the incident optical power. No significant variation in the optical gain was observed over the 4-decade decrease in incoming optical power. This high dynamic range of performance is promising for LWIR applications with photon starvation or very low light levels.

**Fig. 5:**
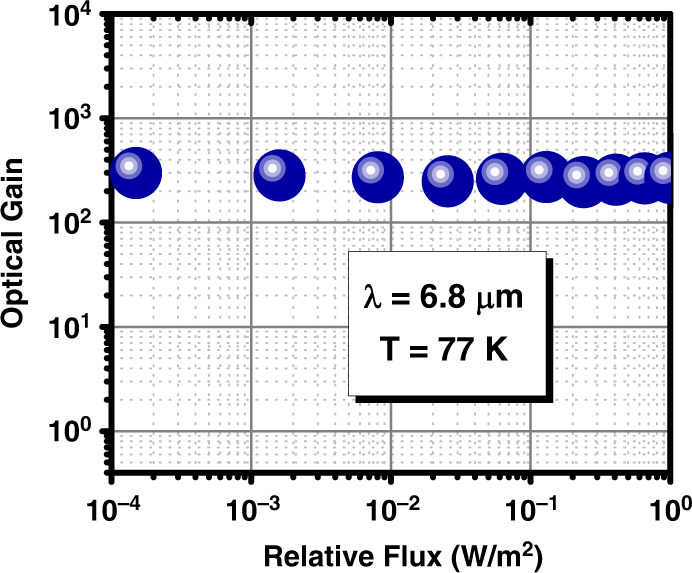
The optical gain of the LWIR phototransistor versus the relative incident optical power at 6.8 μm (*T* = 77 K and *V*_b_ = 220 mV)

With the knowledge of the optical and electrical performance, another important figure of merit is the shot noise limited specific detectivity (D*). The overall performance of a device can be standardized by the specific detectivity. The detective is a parameter defined to characterize the capability of detecting photons of a given detector. This capability is usually described by the ratio between the signal level and the noise level, which compares the photo-generated current to the intrinsic noise level of the device^50,51^. D* can be calculated as follows

1 $$D^ \ast = R_i[2qJ + 4k_BT/R^\ast A]^{ - 1/2}$$

where *R*_*i*_ is the responsivity*, q* is the electronic charge*, J* is the dark current density*, k*_*B*_ is the Boltzmann constant, and R*A is the resistance-area product. The high-gain LWIR photodetector shows a D* of 2.34 × 10^13^ cm Hz^1/2^/W under saturated bias (*V*_b_ = 220 mV) at *T* = 77 K (Fig. 6a). This device demonstrates a high specific detectivity value across a broad range of wavelengths. To better understand the relative performance of the device, Fig. 6a compares the detectivity spectrum of this device with our previous report for a heterojunction T2SL-based LWIR n-contact-barrier-n-contact (*nBn)* detector^52^ with a similar active region thickness and a comparable cut-off wavelength. At 77 K, the high-gain LWIR device presented in this paper exhibits a saturated specific detectivity that is almost 30 times higher than that of the comparable *nBn* detector. The higher specific detectivity for the same incoming optical power can offer major advantages for highly sensitive LWIR detectors. The heterojunction *nBn* structure is incapable of providing any sort of gain with limited QE, whereas the band-structure-engineered LWIR device is able to deliver high responsivity with a very high gain. As shown by implementing the band structure engineering approach for the LWIR photodetector in the present work, for the same incoming optical power, a much higher level of detectivity than that of the heterojunction *nBn* structure can be achieved.

**Fig. 6:**
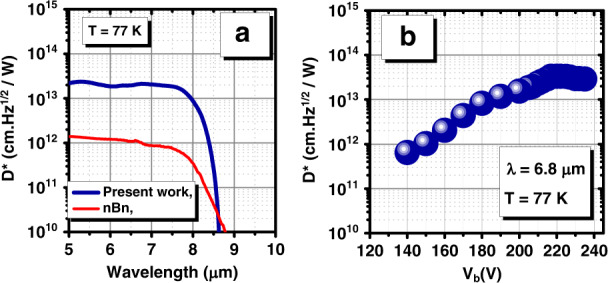
Specific detectivity performance of the LWIR T2SL phototransistor. **a** specific detectivity spectrum comparison as a function of the wavelength at *T* = 77 K between the LWIR T2SL phototransistor device under *V*_b_ = 220 mV and a T2SL-based LWIR heterojunction (*nBn*) detector at *V*_b_ = 100 mV in the front-side illumination configuration without any anti-reflection coating. **b** Specific detectivity variation vs. applied bias at 6.8μm and 77K

In addition, because of the two-contact design and modest bias requirements, this design is compatible with existing read-out integrated circuits (ROICs) and could be used to realize improved LWIR imaging in infrared camera applications^53–55^. The D* values remain approximately unchanged across a certain range of bias voltages.

If the applied bias is reduced from 220 mV, the gain decreases, and the specific detectivity (D*) also decreases (Fig. 6b). However, at any applied bias between 150 and 220 mV, the specific detectivity still exceeds that of the reference device. This allows trading reduced dark current density for lower optical gain, which may be necessary to create an image with this design if the full well capacity of the ROIC to integrate the signal and dark current is limited.

In conclusion, using the band structure tunability of a type-II superlattice material system, we have reported the design, growth, and characterization of high-gain LWIR photodetectors. At the peak detection wavelength of ~6.8 µm, the device responsivity saturates at 1284 A/W under 220 mV of applied bias. The 1/*e* cut-off wavelength of the device is 8.0 µm. Unity optical gain occurs at a 90 mV applied bias with a dark current density of 3.2 × 10^−7^ A/cm^2^. The optical gain saturates at 276 at an applied bias of 220 mV; at this bias, the current gain is estimated to be 833. Further enhancement of the performance by scaling the base thickness could be considered a possible direction^56^. The type-II superlattice-based high-gain LWIR device presented in this work presents a new possibility for the design of high-gain LWIR photodetectors. These devices may also be suitable for focal plane array imaging and for high-speed applications such as light detection and ranging if the bandwidth is engineered by scaling the emitter–base junction along with carefully grading the base–emitter heterojunction to control the capacitance and recombination rate. Use of a wide-wavelength multi-layered antireflection coating on top of the device can also be implemented to enhance the optical performance of the device for future applications.

## Materials and methods

The material for the high-gain LWIR T2SL phototransistor was grown on a Te-doped *n*-type (*n* ~ 10^17^ cm^−3^) GaSb wafer in an Intevac GEN-II solid-source MBE (SSMBE) reactor. The details of the growth of the structure are given in the supporting information. Growth was started with an InAs_0.91_Sb_0.09_ template layer, and then the device structure was grown (sectors 1–7). Beryllium (Be) and silicon (Si) were used to achieve p-type and n-type doping, respectively, while the temperature of the Si and Be cells in the SSMBE was controlled to generate different doping concentration levels. Following the MBE epitaxial growth, high-resolution X-ray diffraction (HR-XRD) and atomic force microscopy were used to evaluate the material quality (details in the Supporting information).

The material was then processed into two-terminal mesa-isolated devices (Fig. 1) with diameters ranging from 100 to 400 μm. The arrows at the right of Fig. 1 show the mesa etching and metallization processes. The devices were left unpassivated, but efforts were made to carry out many surface cleaning steps to minimize the amount of surface leakage.

Specific details about the device processing steps are given in the supporting information. Several samples of the LWIR T2SL photodetector with the same structure and design were grown and tested to ensure the reproducibility of the results.

After fabrication, the LWIR T2SL device was wire-bonded to a 68 pin leadless ceramic chip carrier and loaded into a Janis cryostat for optical and electrical testing at a cryogenic temperature of 77 K.

## Supplementary information

Supplementary Information

## Data Availability

The data that support the findings of this study are available from the corresponding author upon reasonable request.
