# Supplementary material for: Band-structure-engineered high-gain LWIR photodetector based on a type-II superlattice
Source: Light Sci Appl. 2021 Jan 14;10:17. doi: 10.1038/s41377-020-00453-x (PMC7809042; doi:10.1038/s41377-020-00453-x)
Supplement: Supplementary file 1 — Supplementary Information [file 41377_2020_453_MOESM1_ESM.docx]

**Supplementary Information for band structure engineered high gain LWIR photodetector based in type II superlattice**

Arash Dehzangi^1^, Jiakai Li^1^, Manijeh Razeghi^1^

^1^Center for Quantum Devices, Electrical Engineering and Computer Science Department, Northwestern University, 2220 Campus Drive, Evanston, IL, USA 60208-0893

**Main contents**

**1. Growth of the LWIR photodetector based on Type-II superlattice (T2SL) materials**

**2. Characterization of the material (High resolution X-ray and atomic force microscope)**

**3. Fabrication of the device**

**4- Current time characterization**

**1. Growth of the LWIR photodetector based on Type-II superlattice (T2SL) materials**

The material for high gain LWIR photodetector was grown on a Te-doped *n*-type (10^17^ cm^−3^) GaSb wafer in an Intevac GEN-II solid-source molecular beam epitaxy (SSMBE) reactor. First, a 100 nm GaSb buffer layer to stabilize the wafer surface and after that a 0.5 μm *n*-doped InAs_0.91_Sb_0.09_ etch stop layer (10^18^ cm^-3^) were grown on the GaSb wafer. Then 7/1/5/1 mono-layers (MLs) of InAs/GaSb/AlSb/GaSb T2SL structure with 0.5 μm thickness *n-type* (10^18^ cm^-3^) bottom contact layer was grown. At the next step, 0.5 μm thick *n-doped* (10^17^ cm^-3^) emitter similar to the bottom contact with 7/1/5/1 MLs of InAs/GaSb/AlSb/GaSb, respectively, was grown. The growth was followed by 60 nm thick *p-doped* (~1×10^16^ cm^-3^) base layer, which consists of 6.5/10 MLs of InAs/GaSb. By reducing the n-type doping level we can generate a thick emitter–base depletion region at the emitter and diminish the depletion capacitance. After growing the supperlattice base, the hybrid collector structure was grown. In hybrid collector, the 0.25 μm thick MWIR was kept undoped but had a residual lightly *n-type* background with concentration around 10^15^ cm^-3^ and superlattice design with 6.5/10 MLs of InAs/GaSb. After that, an *n-type* (10^16^ cm^-3^) 1.0 μm collector with the 13/7 MLs of InAs/GaSb, was grown.

The growth followed by growing another n-type (10^18^ cm^-3^) 0.25 μm hole barrier with the superlattice design, 18/3/4/3 MLs of InAs/GaSb/AlSb/GaSb, was grown. Finally, an *n-type* (10^18^ cm^-3^) 0.2 μm-thick top contact layer with the superlattice design, 13/10 MLs of InAs/GaSb was grown on top of the structure. Silicon was used as the n-type dopant, and beryllium was used as the p-type dopant during growth.

**2. Characterization of the material (High resolution X-ray and atomic force microscope)**

After the epitaxial growth, the material quality was assessed using atomic force microscopy (AFM) and high resolution X-ray diffraction (HR-XRD). Figure S1 shows the structural characterization results of as grown material for bandstructure-engineered LWIR photodetector. The sample exhibited a good surface morphology with clear atomic steps and small roughness of 1.01 A˚ over a 5 × 5 μm^2^ area (Figure S1a). The HR-XRD was well-fitted to the simulation result (Figure S1b). There is no sign of relaxation or dislocation, meaning a good crystallinity.


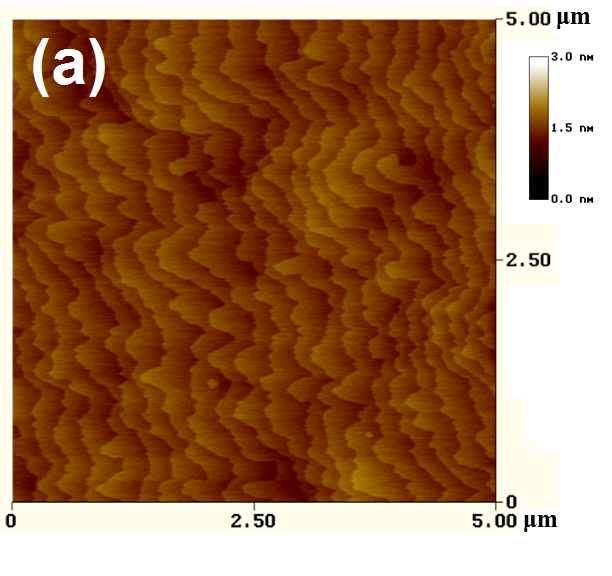

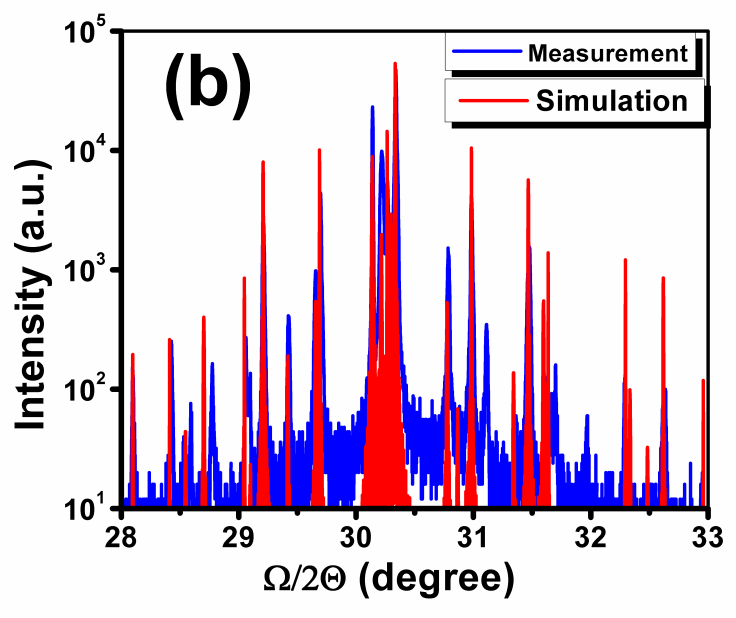


**Figure S1.** (a) The AFM image of a 5 μm × 5 μm surface area of the as-grown material, with rms roughness value of 1.01 Å. (b) HR-XRD curve and simulation of the material grown for the LWIR device

**3. Fabrication of the device**

After growth samples were treated with simple cleaning and degreasing. The processing can be divided into two main steps, mesa isolation and metal contacts deposition. First, photolithography was performed on top of the sample to create the photoresist pattern for mesa etching. For the etching process combination of dry and wet etching were applied. To perform the dry etching, an inductive couple plasma reactive ion etching (ICP-RIE) (Oxford Plasma Lab System) was implemented. This system permits to use Chlorine (Cl) or Fluorine (F) based plasmas, which we selected to use a combination of BCl_3_ and Ar to attain low etching rates (0.2 μm.min^-1^). To get smoother sidewalls major parameters involved in dry etching (e.g. temperature, pressure) were optimized. During dry etching, the photoresist starts being attacked by the plasma, which can create some deformations along the vertical directions on the sidewalls. Moreover, the superlattice in the vicinity of the sidewalls can be subjected to ICP-RIE aggressive process. In order to smoothen the sidewalls, having more uniform etching, and remove the damaged materials, we shortly wet etched the superlattice right after dry etch, using citric acid base isotropic wet etching. After the etching step, solvent base cleaning was performed to remove all the impurities, etching residues and surface contaminants. Most of the contamination is due to photoresist residues left after the etching or metallization. AZ KWIK strip remover at high temperatures (100°C) was used to clean the photoresist after each photolithography, and then complementary cleaning was applied using high purity solvents (acetone, methanol, and propanol). After etching, a CHS electron beam evaporator was used to deposit Ohmic metal contact (Ti/Au stack) for the top and bottom contacts of the photo detector.


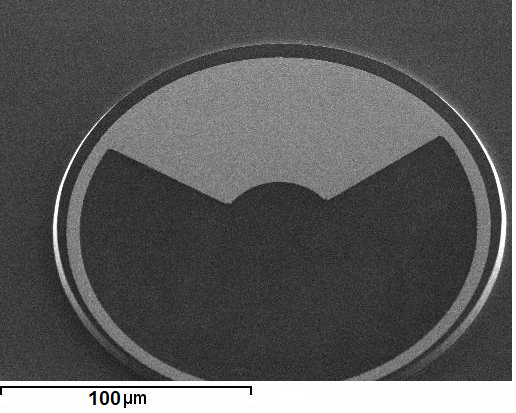


**Figure S2.** Scanning electron microscope (SEM) image of processed single element devices

During the metallization, to ensure uniform thickness of metal layers, the evaporation rate was controlled (<2 Å/sec) and the deposition thickness was monitored in real-time by a quartz crystal. After metal deposition and lift-off, again solvent cleaning were performed to prepare the sample for testing. The samples were processed into mesa-isolated single element diodes, the sizes vary from 100×100 μm^2^ to 400×400 μm^2^. No specific passivation procedure was performed here but extensive percussion is taken to minimize the surface leakage. After the processing, the samples are wire-bonded onto a leadless ceramic chip carrier (LCCC) and loaded into a cryostat measurement system for optical and electrical testing.

**4.** **Current-Time characterization**

In order to determine the reproducibility of the results over time Current-Time characterization was performed. The dark current density as a function of time for the LWIR HPT sample is illustrated in Figure S3. The applied bias voltage was chosen to be at the middle range of operation V_b_ = 180 mV with the corresponding an operating dark current density of 1.86×10^-4^ A/cm^2.^. Over continuous test over the period of 100 hours for the sample no major fluctuation, increase or degradation was observed.


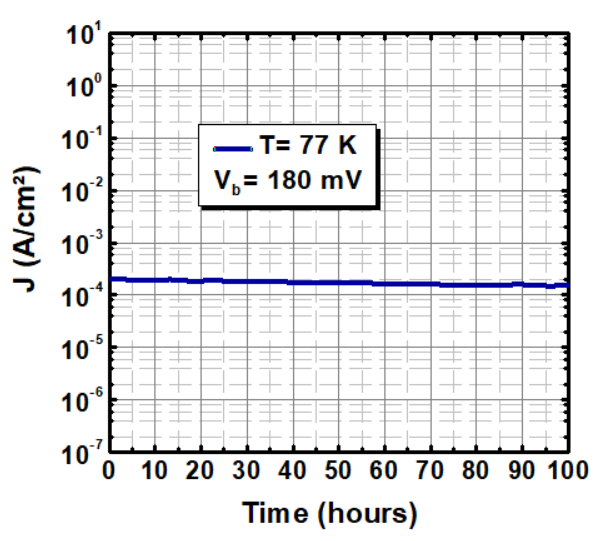


**Figure S3.** The Dark current density output of LWIR T2SL HPT as a function of time at 77 K at a constant bias voltage of 180 mV.
